# Bronchoalveolar Lavage Enzyme-Linked Immunospot for Diagnosis of Smear-Negative Tuberculosis in HIV-Infected Patients

**DOI:** 10.1371/journal.pone.0039838

**Published:** 2012-06-26

**Authors:** Adithya Cattamanchi, Isaac Ssewenyana, Rose Nabatanzi, Cecily R. Miller, Saskia Den Boon, J. Lucian Davis, Alfred Andama, William Worodria, Samuel D. Yoo, Huyen Cao, Laurence Huang

**Affiliations:** 1 Division of Pulmonary and Critical Care Medicine, San Francisco General Hospital, University of California San Francisco, San Francisco, California, United States of America; 2 Joint Clinical Research Centre, Kampala, Uganda; 3 Makerere University-UCSF Research Collaboration, Kampala, Uganda; 4 Department of Medicine, Makerere University, Kampala, Uganda; 5 Division of Infectious Diseases, University of California San Francisco, San Francisco, California, United States of America; 6 HIV/AIDS Division, San Francisco General Hospital, University of California San Francisco, San Francisco, California, United States of America; University of Cape Town, South Africa

## Abstract

**Background:**

Peripheral blood interferon-gamma release assays (IGRAs) have sub-optimal sensitivity and specificity for diagnosis of active pulmonary tuberculosis (TB). However, assessment of local immune responses has been reported to improve the accuracy of TB diagnosis.

**Methods:**

We enrolled HIV-infected adults with cough ≥2 weeks’ duration admitted to Mulago Hospital in Kampala, Uganda and referred for bronchoscopy following two negative sputum acid-fast bacillus smears. We performed an ELISPOT-based IGRA (T-SPOT.*TB*®, Oxford Immunotec, Oxford, UK) using peripheral blood and bronchoalveolar lavage (BAL) fluid mononuclear cells, and determined the accuracy of IGRAs using mycobacterial culture results as a reference standard.

**Results:**

94 HIV-infected patients with paired peripheral blood and BAL IGRA results were included. The study population was young (median age 34 years [IQR 28–40 years]) and had advanced HIV/AIDS (median CD4+ T-lymphocyte count 60 cells/µl [IQR 22–200 cells/µl]). The proportion of indeterminate IGRA results was higher in BAL fluid than in peripheral blood specimens (34% vs. 14%, difference 20%, 95% CI 7–33%, p = 0.002). BAL IGRA had moderate sensitivity (73%, 95% CI 50–89%) but poor specificity (48%, 95% CI 32–64%) for TB diagnosis. Sensitivity was similar (75%, 95% CI 57–89%) and specificity was higher (78%, 95% CI 63–88%) when IGRA was performed on peripheral blood.

**Conclusions:**

BAL IGRA performed poorly for the diagnosis of smear-negative TB in a high HIV/TB burden setting. Further studies are needed to examine reasons for the large proportion of indeterminate results and low specificity of BAL IGRA for active TB in high HIV/TB burden settings.

## Introduction

Interferon-gamma release assays (IGRAs) are being used increasingly for the detection of *Mycobacterium tuberculosis* (MTB) infection. Commercial ELISA- and ELISPOT-based assays that detect interferon-gamma release following overnight stimulation with MTB-specific antigens (Early Secreted Antigen Target-6 [ESAT-6] and Culture Filtrate Protein-10 [CFP-10]) are now recommended as an alternative to the tuberculin skin test for targeted testing of individuals for latent tuberculosis (TB) infection. [1], [2] However, the diagnostic accuracy of blood IGRAs for active TB has been disappointing, with sub-optimal sensitivity, especially among the immunocompromised, and low specificity, especially in high TB incidence areas, identified as particular concerns. [3], [4].

Assessing MTB-specific immune responses at the site of disease (*i.e.*, the lungs) may improve IGRA performance for diagnosis of active pulmonary TB. T-cells secreting IFN-γ following stimulation with either purified protein derivative (PPD) or *M. TB*-specific antigens (ESAT-6 and CFP-10) have been detected at 10- to 100-fold higher frequency in bronchoalveolar lavage (BAL) fluid compared to peripheral blood specimens in patients with pulmonary TB [5], [6], which may enhance the sensitivity of IGRAs. In addition, a lower ratio of central memory T-cells to effector T-cells in the lung may increase the specificity of immunologic assays for active disease. [7] In support of these hypotheses, a prospective, multi-center study of 347 HIV-uninfected TB suspects in Europe found BAL IGRA superior to blood IGRA, the tuberculin skin test, and nucleic acid amplification tests for the diagnosis of smear-negative pulmonary TB. [8].

Although bronchoscopy with BAL is unavailable in many TB and HIV endemic settings, where it is available, it does improve TB diagnosis [9] and BAL IGRA could further increase its yield. Thus, we conducted a prospective study to evaluate the diagnostic accuracy of BAL IGRA, and to compare BAL to peripheral blood IGRA, in a country with high TB and HIV prevalence.

## Methods

### Ethics Statement

Institutional review boards at Makerere University, Mulago Hospital, the Uganda National Council for Science and Technology, and the University of California, San Francisco approved the study protocol, and all patients provided written informed consent for study participation.

### Study Population

We prospectively enrolled consecutive HIV-infected patients with suspected pulmonary TB who were sputum acid-fast bacillus (AFB) smear-negative and referred for bronchoscopy at Mulago Hospital in Kampala, Uganda. We excluded patients from the analysis if TB status could not be established due to mycobacterial culture contamination (at least two negative cultures were required to exclude TB) or if blood or BAL IGRA testing was not performed.

### Patient Evaluation and Sample Collection

We administered a standardized questionnaire and reviewed hospital records to collect demographic and clinical information, including confirmation of HIV status recorded through the Mulago Hospital voluntary counseling and testing program. We collected blood samples for IGRA testing and measurement of CD4+ T-lymphocyte count and two sputum samples for Lowenstein-Jensen culture, as previously described. [10] Bronchoscopy was performed for clinical indications and patients were consented in accordance with standard clinical protocols. Bronchoscopy was performed by clinical investigators (WW, SY, AC, JLD) according to a standardized protocol that included collection of BAL fluid. [11] Briefly, bronchoscopists performed BAL by wedging the tip of the bronchoscope into the sub-segment of lung with the greatest radiographic abnormality, instilled sterile normal saline (0.9%) in serial 25 mL aliquots (up to a maximum of 125 mL), and aspirated fluid until at least 50 mL were returned. Study technicians immediately prepared at least 20 mL of BAL fluid for IGRA testing, as described below.

### IGRAs

Trained laboratory technicians at the Joint Clinical Research Centre (JCRC) blinded to patients’ clinical status performed blood and BAL IGRAs. Peripheral blood mononuclear cells (PBMCs) were isolated using Ficoll-hypaque gradient centrifugation. BAL fluid mononuclear cells (BALMCs) were obtained by passing the sample through a 50 micron nylon mesh filter, pelleting the sample by centrifugation (450×G for 10 minutes), and re-suspending the pellet in R10 culture medium. PBMC and BALMC count and viability were determined using a Guava automated counter (Guava Technologies, Hayward, CA). Blood and BAL IGRAs were performed using a commercially-available, enzyme-linked immunospot (ELISPOT)-based assay (T-SPOT.*TB*®, Oxford Immunotec, Oxford, UK) according to the manufacturer’s guidelines for peripheral blood assays. Spot-forming cells (SFCs) were counted using an automated ELISPOT reader (Immunospot Analyzer, Cellular Technologies, Ltd. Cleveland, OH).

For the primary analysis, we interpreted peripheral blood IGRA results according to manufacturer-recommended criteria and BAL fluid IGRA results according to criteria published by the Tuberculosis Network European Trials Group (TB NET). [8] We also performed secondary analyses in which we classified BAL IGRA results using alternative classification schemes that did not exclude samples with high background interferon-gamma secretion. First, as reported for in-house assays, we classified BAL IGRA results as positive if the number of SFCs in either antigen-stimulated well was at least two standard deviations above the background control plus 5 SFCs. [12] Next, we analyzed BAL IGRA results based on the ratio of SFCs between antigen-stimulated wells and the background control well plus 5 SFCs.

### Outcome Classification

We defined the reference standard for active TB as at least one positive mycobacterial culture result on any sputum or BAL fluid specimen (culture-positive TB). Cultures were interpreted without knowledge of IGRA results.

### Statistical Analysis

We performed bivariate analyses using the Fisher’s exact test for dichotomous variables and the Mann-Whitney rank-sum test for continuous variables. We compared median SFC counts between BAL and blood IGRAs using the Wilcoxon matched-pairs signed-ranks test. We calculated sensitivity and specificity of IGRAs using mycobacterial culture results as a reference standard, and compared sensitivity and specificity differences between BAL and blood IGRAs using McNemar’s test. We determined the area under the curve (AUC) using receiver operating characteristic (ROC) analysis to evaluate the diagnostic utility of BAL IGRA at various cut-points based on the ratio of SFCs in the antigen-stimulated wells and the negative control well. We performed all analyses using STATA 11.0 (Stata Corporation, College Station, Texas), with the level of significance specified in reference to a two-tailed, type I error (p-value) less than 0.05.

## Results

### Study Population

**Figure 1:**
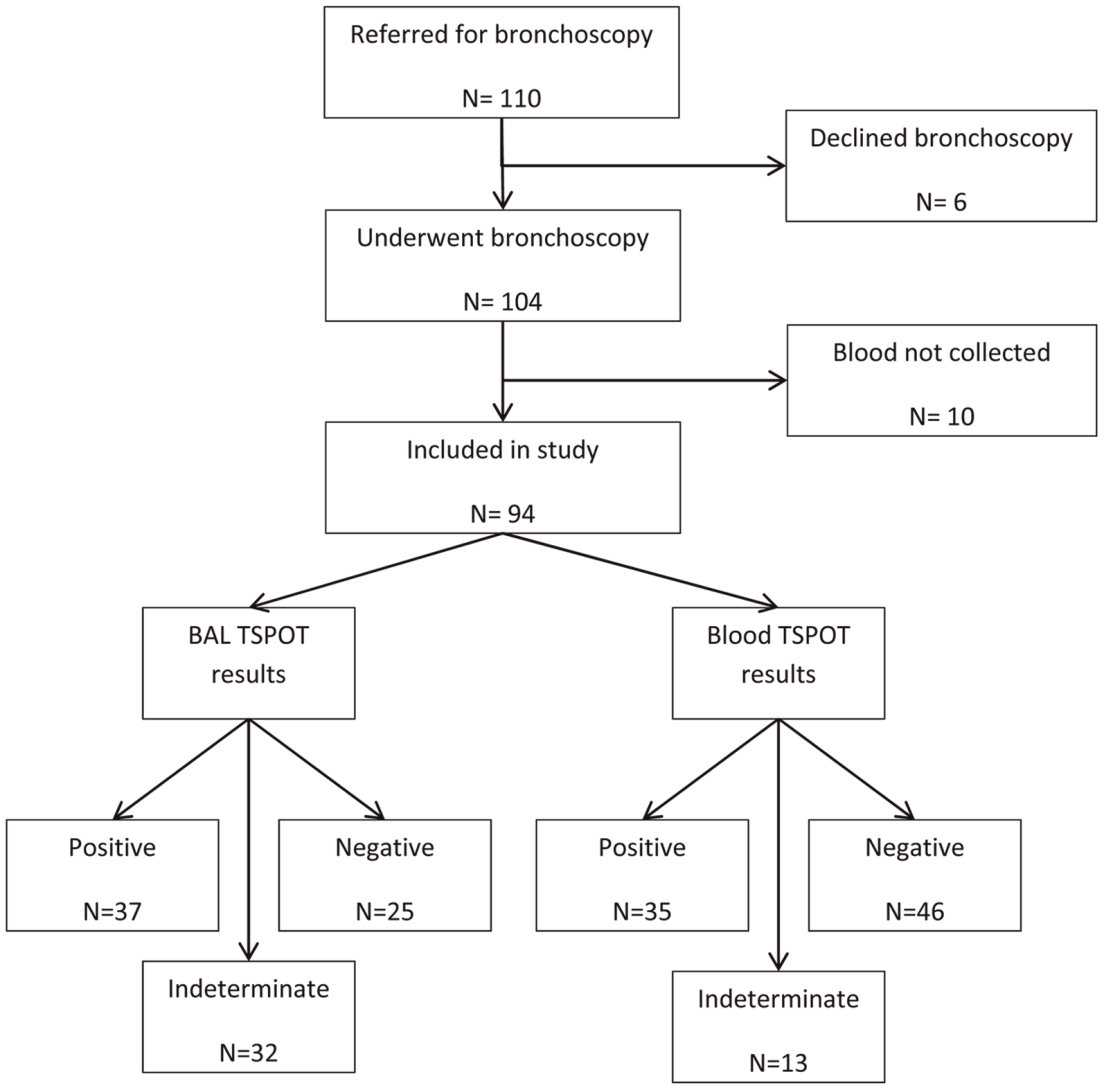
Study flow. Of 110 smear-negative tuberculosis suspects referred for bronchoscopy, 104 (95%) underwent the procedure and 94 (85%) were included in the study. Bronchoalevolar lavage TSPOT and peripheral blood TSPOT provided interpretable results in 62 (66%) and 81 (86%) patients, respectively.

Of 110 HIV-infected patients referred for bronchoscopy between June 2009 and December 2010, 6 patients who declined the procedure and 10 patients for whom blood samples were not collected were excluded from the analysis (Figure 1). Baseline characteristics (gender, age, antiretroviral use, and CD4+ T-lymphocyte count) were not significantly different between excluded and included patients (p>0.2 for all comparisons). Of the 94 included patients, 50 (53%) were women and the median age was 34 years (IQR 28–40 years) (Table 1). The study population had advanced HIV/AIDS (median CD4+ T-lymphocyte count 60 cells/µl [IQR 22–200 cells/µl]), but only 15 (16%) patients were receiving anti-retroviral therapy. The reference standard outcome of culture-positive TB was confirmed in 36 (38%) patients, of whom 5 only had a positive BAL fluid culture result.

**Table 1:** Demographic and clinical characteristics.

Characteristic, N%	Total (N = 94)
Age (years)*	34 (28–40)
Female	50 (53%)
Fever	85 (90%)
Weight loss	86 (92%)
Hemoptysis	13 (15%)
Prior active tuberculosis	7 (7.5%)
CD4+ T-lymphocyte count (cells/µl)*	60 (22–200)
Anti-retroviral use	15 (16%)
TB culture-positive	36 (38%)

* Continuous variables presented as medians (interquartile range).

**Abbreviations:** TB (tuberculosis).

### IGRA Results

BAL IGRAs were indeterminate in 32 (34%) patients (Table 2). Of the 32 indeterminate results, 13 had positive control wells with ≤20 SFCs (after subtraction of background), 13 had positive control wells with less than twice the number of SFCs in the negative control well, and 6 met both criteria for an indeterminate result. The proportion of indeterminate results was similar among TB and non-TB patients (39% vs. 32%, p = 0.50). In addition, there were no differences in gender, age, CD4+ T-lymphocyte count, anti-retroviral use, or in-hospital mortality in patients with and without indeterminate BAL IGRAs (p>0.2 for all comparisons). BAL IGRAs were positive in 37 of 62 (60%) patients with interpretable results. The proportion of positive BAL IGRAs was similar among patients with a CD4+ T-lymphocyte count ≤50 cells/µl (15/27, 66%), 51–200 cells/µl (11/18, 61%), and >200 cells/µl (10/15, 67%) (p = 0.80). Median SFC counts were significantly higher in patients with active TB compared to those without active TB after ESAT-6 (114 vs. 7, p = 0.049) but not CFP-10 (6 vs. 9, p = 0.89) stimulation.

**Table 2:** BAL and blood IGRA results (N = 94).

Blood IGRA	BAL IGRA		
	Negative	Positive	Indeterminate
Negative	18	13	15
Positive	5	17	13
Indeterminate	2	7	4

**Abbreviations:** BAL (bronchoalveolar lavage), IGRA (interferon-gamma release assay).

Blood IGRAs were less often indeterminate than BAL IGRAs (14% vs. 34%, difference −20%, 95% CI −33% to −7%, p = 0.002), and 4 of 13 patients with indeterminate blood IGRAs also had indeterminate BAL IGRAs (Table 2). Blood IGRAs were positive in 35 of 81 (43%) patients with interpretable results, including 17 of 37 (46%) patients with positive BAL IGRAs. In 53 patients with interpretable results for both assays, median SFC counts following ESAT-6 stimulation were higher in BAL IGRAs than in blood IGRAs overall (11 [IQR 0–167] vs. 1 [IQR 0–16], p = 0.003) and among non-TB patients (3 [IQR 0–90] vs. 0 [IQR 0–2], p = 0.001). However, among TB patients, median ESAT-6 SFC counts were not significantly different for BAL and blood IGRAs (84 [IQR 0–402] vs. 25 [IQR 1–245], p = 0.30). The same patterns were observed following CFP-10 stimulation, with significant differences in SFC counts between BAL and blood IGRAs overall (5 [IQR 0–74] vs. 0 [IQR 0–7], p = 0.006) and among non-TB patients (6 [IQR 0–103] vs. 0 [IQR 0–0], p<0.001), but no significant differences between BAL and blood IGRAs among TB patients (4 [IQR 0–48] vs. 7 [0–69], p = 0.90).

### Diagnostic Performance

BAL IGRA sensitivity was 73% (95% CI 50–89%) and specificity 48% (95% CI 32–64%) in 62 patients with interpretable results (Table 3). Blood IGRA had similar sensitivity (75%, 95% CI 57–89%) but higher specificity (78%, 95% CI 63–88%) in 81 patients with interpretable results (Table 4). The specificity difference was statistically significant (24% vs. 76%, difference −52%, 95% CI −76% to −30%, p<0.001) in 34 non-TB patients with interpretable results for both assays. In addition, specificity was similar (56%, 95 CI 30%–80%) when only 16 patients with a confirmed alternative diagnosis (2 with *Pneumocystis jirovecii* pneumonia, 2 with pulmonary kaposi’s sarcoma, and 12 with bacterial pneumonia) were included in specificity calculations.

**Table 3:** Diagnostic accuracy of BAL IGRA.

BAL IGRA	Culture-positive TB	
	Yes	No
Positive	16	21
Negative	6	19
Sensitivity*	73% (50–89%)	
Specificity*	48% (32–64%)	
PPV*	43% (27–61%)	
NPV*	76% (55–91%)	

* **% (95% confidence interval); indeterminate IGRA results excluded.**

**Abbreviations:** BAL (bronchoalveolar lavage), IGRA (interferon-gamma release assay), TB (tuberculosis), PPV (positive predictive value), NPV (negative predictive value).

**Table 4:** Diagnostic accuracy of peripheral blood IGRA.

Blood IGRA	Culture-positive TB	
	Yes	No
Positive	24	11
Negative	8	38
Sensitivity*	75% (57–89%)	
Specificity*	78% (63–88%)	
PPV*	69% (51–83%)	
NPV*	83% (69–92%)	

* **% (95% confidence interval); indeterminate IGRA results excluded.**

**Abbreviations:** IGRA (interferon-gamma release assay), TB (tuberculosis), PPV (positive predictive value), NPV (negative predictive value).

Classification of results based on alternative cut-points did not improve the diagnostic accuracy of BAL IGRA. When using a cut-point reported for non-commercial ELISPOT-based IGRAs (>2 standard deviations plus 5 SFU above background), BAL IGRA was negative in 74 (79%) patients. Sensitivity was 31% (95% CI 16–48%) and specificity 85% (95% CI 73–93%). BAL IGRA also had poor discriminatory ability when results were classified based on the ratio of SFCs in antigen-stimulated and background wells (AUC 0.64, 95% CI 0.52–0.75).

## Discussion

In this study, we found that BAL IGRA had similar sensitivity but lower specificity than blood IGRA, and diagnostic accuracy did not improve with alternate schemes for classification of results. In addition, approximately one-third of BAL IGRA results were indeterminate, a significantly higher proportion than for blood IGRAs. These findings suggest that BAL IGRA performed using the currently available commercial ELISPOT-based platform is unlikely to have a role in the diagnosis of smear-negative TB in hospitalized patients with advanced HIV/AIDS, the population most likely to have access to such testing in high burden countries.

Our results differ from previous studies of BAL IGRA, which have largely been conducted in Europe and included mainly ambulatory patients without HIV infection. In these studies, Jafari et al reported that the sensitivity of BAL IGRA ranged from 91–100% and specificity from 80–100%. [8], [13], [14] Moreover, indeterminate results were relatively infrequent, occurring in 5–9% of patients. Potential reasons for the discrepant findings between these studies and ours include differences in study setting (low- vs. high-incidence countries), study population (HIV status and severity of illness), and relative inexperience with performing BAL IGRA. In the only other study from a high burden country, Dheda et al found BAL IGRA to have high sensitivity (89%) and specificity (94%) using a cut-point selected based on ROC analysis, but results were indeterminate in 34% of patients. [15] Reasons for the lower specificity observed in our study are unclear but are consistent with a study reporting detection of *M. tuberculosis* antigen-specific T-cells in the BAL fluid of individuals without active tuberculosis in another high TB burden country. [16] Thus, as for blood IGRAs, the lower specificity of BAL IGRAs for active TB in high vs. low TB incidence settings is not surprising. Similarly, the high proportion of indeterminate BAL IGRA results in our study and that of Dheda et al is consistent with the higher proportion of indeterminate results observed in high vs. low TB incidence settings when IGRAs have been applied to pleural fluid specimens. [17], [18], [19].

The large proportion of indeterminate results observed in high burden countries poses a challenge for a lung-oriented approach to immunodiagnosis of active pulmonary TB. Based on the criteria used to categorize blood assays, 19 of 32 (59%) indeterminate BAL IGRAs in our study could be considered to have a high background response. For these 19 cases, there were minimal differences in SFC counts between the negative control well and the antigen-specific wells. The high background response could be a result of non-specific immune activation in African populations as result of chronic exposure to helminths, other infectious agents, and/or environmental pollutants. [20], [21], and may also reflect HIV-related immune activation However, the interpretation is limited, as viral load testing was not done and a relationship between viral load and immune activation could thus not be established. In addition, non-specific immune activation alone is unlikely to explain the higher background response observed in BAL relative to blood IGRAs. An alternative explanation may be detection of interferon-gamma secretion by terminally differentiated effector T-cells rather than by MTB-specific effector T-cells. The accumulation of terminally differentiated effector T-cells is a hallmark of early immune senescence in advanced HIV/AIDS [22], and phenotypic studies have suggested that terminally differentiated effector T-cells may be concentrated at the site of disease in patients with active TB. [23] Further studies are needed, as high background responses may represent a potential Achilles heel for local immunodiagnosis of active TB among patients with HIV/AIDS living in TB endemic settings.

Our study has several potential limitations. First, our study population consisted of severely ill, hospitalized patients with advanced HIV/TB, who may not be representative of the general population of TB suspects in high-incidence areas. Nevertheless, our findings are applicable to referral hospitals in sub-Saharan Africa – the settings most likely to provide bronchoscopy services in low-income countries. Second, mycobacterial culture on Lowenstein-Jensen media is an imperfect reference standard. A more sensitive culture technique (such as liquid culture) and clinical follow-up may have more accurately classified TB status and therefore increased the sensitivity and specificity of BAL IGRA. [24] Finally, the poor performance of BAL IGRA in our study could be related to technical issues with processing BAL fluid specimens. The JCRC laboratory that performed this work is the central laboratory for the National Institutes of Health (NIH)-funded HIV Vaccine Prevention Trials Network, and JCRC technicians have more than 10 years of experience performing ELISPOT assays. Nonetheless, methods for processing BAL fluid specimens for ELISPOT assays should be standardized to improve comparability of results across studies.

In summary, BAL IGRA performed poorly for the diagnosis of smear-negative pulmonary TB in a hospitalized population with advanced HIV/AIDS. Sensitivity and specificity were inadequate, and a large proportion of results were indeterminate, mainly due to high background secretion of interferon-gamma. Further research on alternative test platforms, biomarkers, and/or antigens is needed to improve diagnostic accuracy and minimize inconclusive results when using a lung-oriented approach to immunodiagnosis of active pulmonary TB in high burden settings.
